# Decavanadate Compound Displays In Vitro and In Vivo Antitumor Effect on Melanoma Models

**DOI:** 10.1155/bca/6680022

**Published:** 2025-01-11

**Authors:** Amine Essid, Ines Elbini, Regaya Ksiksi, Nardine Harrab, Wassim Moslah, Imen Jendoubi, Raoudha Doghri, Mohamed-Faouzi Zid, José Luis, Najet Srairi-Abid

**Affiliations:** ^1^Institut Pasteur de Tunis, LR20IPT01 Biomolécules, Venins et Application Théranostiques (LBVAT), University of Tunis El Manar, Tunis 1002, Tunisia; ^2^Faculty of Sciences of Tunis, Laboratory of Materials, Crystal Chemistry and Applied Thermodynamics, University of Tunis El Manar, El Manar II, Tunis 2092, Tunisia; ^3^Laboratoire de Médecine de Précision, Médecine Personnalisée et Investigation en Oncologie (LR21SP01), Service d'Anatomie Pathologique, Institut Salah Azaiez, Bab Saadoun, Tunis 1006, Tunisia; ^4^Institut de Neurophysiopathologie, INP, Faculté des Sciences Médicales et Paramédicales, CNRS, Aix-Marseille Université, Marseille 13005, France

**Keywords:** antitumor agents, cancer inhibitor, cell migration, decavanadate, melanoma, polyoxometalates, polyoxovanadate

## Abstract

The efficacy of available treatments for melanoma is limited by side effects and the rapidly emerging resistance to treatment. In this context, the decavanadate compounds represent promising tools to design efficient therapeutic agents. In our study, we synthesized a dimagnesium disodium decavanadate icosahydrate compound (Mg_2_Na_2_V_10_O_28_·20H_2_O) and investigated its structure stability as well as its antimelanoma effects. Results showed that the Mg_2_Na_2_V_10_O_28_·20H_2_O compound is structured in a monoclinic system with the space group C2/c, stabilized by oxygen vertices, hydrogen bonds, and van der Waals interactions. Interestingly, we found that this newly synthesized compound reduced the viability of human (IGR39, IGR37, and SKMEL28) and murine (B16-F10) melanoma cells in a dose-dependent manner. The IC_50_ values ranged from 7.3 to 18 μM after 24 h and decreased to 1.4 μM after 72 h of treatment. Notably, this effect was more important than that of cisplatin (IC_50_ = 3 μM after 72 h of treatment), a chemotherapeutic agent, commonly used in the treatment of malignant melanoma. Furthermore, the cytotoxicity of the decavanadate compound was relatively weak on normal human keratinocytes (HaCaT), with a light effect (IC_50_ >> 70 μM) observed after 24 h of treatment. Thus, the Mg_2_Na_2_V_10_O_28_·20H_2_O compound displayed an advantage over cisplatin, which was reported to be much more aggressive to the keratinocyte cell line (IC_50_ = 23.9 μM). Moreover, it inhibited dose-dependently the adhesion of IGR39 cells to collagen (IC_50_ = 2.67 μM) and fibronectin, as well as their migration with an IC_50_ value of 2.23 μM. More interestingly, its in vivo administration to B16-F10 allografted mice, at the nontoxic dose of 50 μg (2.5 mg/kg), prevented and suppressed by 70% the tumor growth, compared to the nontreated mice. Moreover, this compound has also allowed a recovery against inflammation induced in mice by a mixture of DMBA and croton oil. Thus, all our results showed the potential of the Mg_2_Na_2_V_10_O_28_·20H_2_O compound to prevent and efficiently treat the growth and metastasis of melanoma.

## 1. Introduction

Melanoma is the most aggressive and severe type of skin cancer, being the cause of almost 75% of deaths in this category [1, 2]. In the last few years, melanoma incidence has risen faster than any other type of cancer in most White populations, with a mean age of about 55 years [3]. The annual incidence increased between 4% and 6% for many European countries, North America, Australia, and New Zealand [4]. According to the American Cancer Society data, almost 100,640 new cases are recorded in 2024 in both genders in the United States, including 59,170 male and 41,470 female cases [5]. The highest rates were reported in Australia and New Zealand (40.3 per 100,000 and 30.5 per 100,000, respectively), then in North America and Northern and Western Europe, while the lowest incidences are recorded in south-central and southeastern Asia (below 0.5 per 100,000) [6].

Malignant melanoma metastasis progresses through a multistep process from invasion, angiogenesis, then extravasation and dissemination, to the colonization of other organs [7]. Every step of this scenario can be rate-limiting and offers potential targets for melanoma therapy. Currently, surgical removal of the tumor remains the only curative treatment of malignant melanoma. In fact, chemotherapy, which is based on alkylating agents such as dacarbazine and temozolomide, is ineffective in treating melanoma [8, 9]. The resistance to therapy and high aggressiveness of the disease limit long-lasting response to treatment and the potential survival of the patient [10]. Hence, the challenge is to develop novel safe drugs that induce apoptosis and suppress the survival pathways of tumor cells.

On these grounds, the anticancer application of metals, including platinum (Pt), lithium (Li), tungstate (W), gold (Au), or vanadium (V), has become an important and a rapidly growing branch of science [11–13]. However, at high concentrations and after prolonged exposure, metal can exhibit toxicity in vivo, leading to damages, such as immune system alterations and genotoxicity [14]. Nevertheless, the combination of organic drugs with metal ions has been reported to develop the effectiveness of the organic moiety [15]. Besides the well-characterized platinum drugs, bioactive metal-based complexes and clusters as well as metal-based nanoparticles have shown demonstrable anticancer activities [16–18].

From these metal complexes, polyoxometalates (POMs) were widely studied during this last decade [19] and showed promising applications in biology [20]. In particular, decavanadate compounds, which belong to the polyoxovanadates (POVs) family, are known for their antitumor activity [21] and their possible biomedical use in the treatment of diabetes [22] and microbial infections [23, 24] via different processes, including apoptosis, cell cycle arrest, changes in metabolic pathways, formation of ROS, and many other cellular mechanisms [25]. The pharmacological effects of decavanadate or vanadium compounds have been attributed mainly to the fact that they have structural and electronic similarities to phosphate [26, 27]. Vanadium species can adopt a stable trigonal bipyramidal geometry similar to that of the phosphate transition state in phosphate-metabolizing enzymes (such as phosphatase alkaline) and inhibit their biological activity [28]. Indeed, several studies using X-ray crystallography and computational methods showed the capacity of decavanadate compounds to interact with different cellular proteins such as phosphatase A, tyrosine kinase receptors, TRPM receptor, myosin, and actin, affecting their functions and generating various biological activities [11]. On the other hand, decavanadate, with high anionic charges, is stabilized by counterions through electrostatic interactions and hydrogen bonds. Actually, decavanadate has been combined with a variety of elements, including protons, alkali-metal ions, and metformin, as well as organic and organometallic cations [29], and has been considered a promising element for medicinal applications, due to its structural properties. Hence, the anticancer effect of decavanadate compounds has been proven on different tumor cell lines [22, 30–32]. However, few studies reported the decavanadate compounds' effects on melanoma [33, 34].

In our previous works, we have synthesized and characterized several decavanadate molecules that belong to different families. We conjugated the V_10_O_28_ with organic bases, alkalis, and alkaline earths [30, 31, 35–54]. The structural studies of these compounds showed that the sodium (Na), potassium (K), and/or zinc (Zn) atoms confer a very high stability to the decavanadate compound. As it happens in the compounds Na_5_·22Li0.78V_10_O_28_·20H_2_O [36], Na_2_[H_4_V_10_O_28_]·14H_2_O [44], Na_2_Li_2_(H_3_O)_2_[V_10_O_28_]·18H_2_O [52], and Na_1.35_K_4.45_Rb_1.20_(H_2_O)_12_(V_10_O_28_)(NO_3_)·3H_2_O [38], where the alkalis form chains of polyhedra, which are connected by pooling the oxygen vertices, edges, and faces, these types of cohesion gave the structures a high stability, which cannot be easily broken.

On the other hand, our structure–activity relationship studies of some compounds led us to demonstrate the value-added of the water molecules in the structure of the decavanadate compounds against the development of cancer cells [50, 53]. For instance, the (C_4_N_2_H_7_)_6_(C_4_N_2_H_6_)_2_V_10_O_28_ compound showed antiproliferative effect on IGR39 cells with an IC_50_ value of 7 μM [53], whereas the Mg(H_2_O)_6_(C_4_N_2_H_7_)_4_V_10_O_28_·4H_2_O had an IC_50_ value of 2 μM [31].

Furthermore, we found that decavanadate compounds containing magnesium (Mg) atoms were more active than those that did not, although the Mg atom forms only octahedrons and the cohesion between the polyhedra of Mg is ensured only by hydrogen bonds and van der Waals interactions. We found that the decavanadate compound containing Mg is the most active on cancer cells [31].

Thus, in this study, we designed and synthesized a highly hydrated inorganic decavanadate compound Mg_2_Na_2_V_10_O_28_·20H_2_O, containing both Mg and Na atoms, with the aim of developing a therapeutic molecule against melanoma, the most invasive and resistant skin cancer.

## 2. Materials and Methods

### 2.1. Decavanadate Compound Synthesis and Structural Characterization

The Mg_2_Na_2_V_10_O_28_·20H_2_O compound was obtained by dissolving vanadium oxide (V_2_O_5_ FLUKA), magnesium hydroxide (Mg(OH)_2_·5H_2_O, Sigma-Aldrich), and sodium hydroxide (NaOH, Sigma-Aldrich) with molar proportions 5:2:2 in 50 mL of distilled water. The mixture has undergone magnetic stirring and heating for about 2 h and then placed in a Petri dish for 5 days at room temperature.

The structure of Mg_2_Na_2_V_10_O_28_·20H_2_O was determined by standard crystallographic methods. Data were collected on an Enraf-Nonius CAD4 diffractometer equipped with graphite monochromatic Mo (K*α*) radiation (*λ* = 0.71073 Å) [55] on a single crystal of suitable size for the structural study, the structure was solved by direct method using the SHELXS-97 program [56], and refinements were carried out by full-matrix least-squares technique on all F2 data using the SHELXL-2014 program [57]. After solving the structure, and according to the bibliography, we determined the chemical formula of the synthesized phase.

The XRD pattern was obtained using a PANalytical “X'PertPro” X-ray diffractometer equipped with a Cu anticathode (CuK*α* radiation, *λ* = 1.54056 Å), at room temperature. The measurement was performed under Bragg–Brentano geometry at 2*θ* with step 0.02° in the 7°–60° range. The experimental diffractogram was recorded in the angular range between 7° and 60° in 2*θ*. For this analysis, a sufficient quantity of the crystals chosen under a polarizing microscope was crushed and then spread uniformly on a glass slide.

The crystallinity and purity of the prepared phase were controlled by comparing the experimental diffractogram of the compound and the calculated one (obtained from data from .cif files using the POWDERCELL program [58]), using the Origin 8.0 software [59].

The IR spectrum of the prepared crystal was obtained in the range of 4000 to 500 cm^−1^ with an FT-IR spectrometer (Nicolet IR 200, USA).

Thermal analysis (TG-DTA) was performed using a LABSYS evo instrument in a temperature range of 25°C–450°C with a scan rate of 5°C/min in an argon atmosphere in circulation.

### 2.2. Cells and Reagents

Murine highly metastatic melanoma (B16-F10) and human melanoma cell lines (SKMEL28, IGR37, and IGR39) as well as skin keratinocyte cell line (HaCaT) were purchased from the American Type Culture Collection (ATCC). Cells were cultured in Dulbecco's modified Eagle's medium (DMEM) or Eagle's minimal essential medium (EMEM) for SKMEL28 cells (Gibco, Sigma), supplemented with 10% fetal bovine serum (FBS), 1% L-glutamine, and 100 IU/mL penicillin/streptomycin. Cells were maintained at 37°C in a humidified atmosphere (5% CO_2_).

7,12-Dimethylbenz[a]anthracene (DMBA) and croton oil were used as initiator and promoter, respectively, of skin carcinogenesis in mice [60]. These products were purchased from Sigma Chemical Co. (St. Louis, MO, USA).

### 2.3. Cell Viability Assessment by 3-(4,5-Dimethylthiazol-2-yl)-2,5-diphenyltetrazolium Bromide (MTT) Assay

The MTT assay was first described as a sensitive, quantitative, and reliable colorimetric assay to measure the viability, proliferation, and activation of cells [61]. Cell viability was assessed at 24 and 72 h of treatment to evaluate cytotoxicity and the effect on cell proliferation of the decavanadate compound, respectively. The concentrations of the decavanadate compound have been determined by weighing the compound in its crystal form and then solubilizing it in distilled water.

All cell lines were seeded in 96-well plates (Nunc, Roskilde, Denmark) at a density of 10^4^ cells per well and were incubated overnight, before treatment with different concentrations of the decavanadate compound. After 24 or 72 h of treatment, fresh media solution containing MTT at 0.5 mg/mL was added to cells and incubated for 3 h at 37°C. The formed formazan crystals were then dissolved in DMSO, and the absorbance was measured at 560 nm using a BioTek EON Microplate Spectrophotometer (Fisher Scientific). Cell viability was expressed as a percentage of the viable cell number of the untreated cells (control [CTR]).

Notably, each experiment was repeated 3 times in triplicate, and the data are presented as the mean ± SD. The concentration of the decavanadate compound required for 50% growth inhibition (IC_50_ value) was estimated as resulting in a 50% decrease in absorbance, compared to the CTR.

### 2.4. Cell Adhesion Assay

Adhesion assays were performed as previously described by Morjen et al. [62]. Briefly, flat-bottom 96-well microtiter plates were coated for 2 h at 37°C with fibronectin or collagen at 10 μg/mL, as extracellular matrix (ECM) proteins, or poly-L-lysine at 50 μg/mL, as a nonspecific attachment substrate. Plates were then washed with PBS and blocked by BSA. IGR39 cells were harvested and resuspended in DMEM, in the presence or absence (CTR) of the tested molecule. After incubation for 30 min at room temperature, cells were added to coated wells in a volume of 50 μL (50,000 cells) and allowed to adhere to the substrate for 1 h at 37°C. Unattached cells were removed by gently washing three times with an adhesion buffer. Residual attached cells were fixed by 1% glutaraldehyde, stained by 0.1% crystal violet, and lysed with 1% SDS. The absorbance was then measured at 600 nm by a microplate reader.

### 2.5. Cell Migration Assay

Wound healing (or scratch) assay is a method to measure two-dimensional cell migration. An artificial gap is generated on a confluent cell monolayer, and the movement is tracked via microscopy or other imaging. Melanoma cells were seeded in 24-well plates at 15 × 10^4^ cells per well and incubated for 24–48 h. After cells reached 90% confluence, wounds were generated using a 10 μL micropipette tip. Media were removed, and the cells were washed with 500 μL PBS and then incubated in a fresh medium containing different concentrations (not showing cytotoxic effect) of the decavanadate compound. After 24 h of treatment, cells were washed with PBS, fixed with cold ethanol, and stained with Giemsa. Images were acquired on an inverted microscope at *T*_0_ and after 24 h (*T*_24_). The percentage of cell migration was calculated using the following formula: cell migration(%)=[(*T*_0_ − *T*_24_))/(*T*_0_)] × 100 where *T*_0_ is the wound area at zero time and *T*_24_ is the wound area after 24 h. All the pictures were taken with a DM-IRE microscope (Leica, Rueil-Malmaison, France) coupled with a digital camera (CCD camera CoolSNAP FX, Princeton Instruments, Trenton, NJ).

### 2.6. Animals

The decavanadate compound Mg_2_Na_2_V_10_O_28_·20H_2_O was assessed for its in vivo toxicity as well as for its eventual therapeutic effect in animal models mimicking melanoma aspects (chemical and allograft models). Adult male C57BL/6 and Swiss albino mice (6–8 weeks old; 20–25 g) were supplied by the animal unit of the Pasteur Institute of Tunis. Animals were kept under a controlled temperature (22°C–25°C), and relative humidity was maintained at 40%–70% with *ad libitum* access to pure water and a standard pellet diet. A photoperiod of 12-h light and dark cycles was maintained.

### 2.7. In Vivo Toxicity/Safety Assessment of Decavanadate Compound

In order to investigate the safety of the Mg_2_Na_2_V_10_O_28_·20H_2_O compound, three doses of 20, 50, and 100 μg were tested on male Swiss albino mice of ∼20 g weight each. Mice were randomly allocated in four groups of eight mice each. These doses correspond to 1, 2.5, and 5 mg/kg, respectively. A stock solution of 7.5 mg/mL (5.5 mM) was first prepared in PBS (1x). Dilutions were done to obtain 3 solutions of 0.2 mg/mL (146 μM), 0.5 mg/mL (366 μM), and 1 mg/mL (732 μM) in PBS, from which a volume of 100 μL was injected to each mouse, corresponding to the three doses. These doses were chosen based on our previous study [46]. Mice of the CTR group received 100 μL of PBS. Decavanadate compound or vehicle was administered daily by intraperitoneal injection for 12 consecutive days in the morning between 09:00 and 10:00 a.m.

Animals were observed individually for general behavior, toxicity symptoms, and mortality during the first 30 min and then daily during the experiment. The body weights were measured at the beginning and at the end of the day. On Day 12, blood samples were drawn from the facial vein of each mouse, using capillary tubes, containing heparin (as an anticoagulant component), for biochemical parameters studies. Mice were then sacrificed by cervical dislocation, and their organs (liver and kidney) were excised. The organs were then fixed in 10% buffered formalin for histopathological examination.

#### 2.7.1. Biochemical Parameters

Plasma samples were obtained by blood centrifugation at 3000 rpm for 10 min and kept at −20°C. The activities of hepatic enzymes, such as aspartate aminotransferase (AST) and alanine aminotransferase (ALT), and creatinine levels were assessed with commercially available diagnostic kits supplied by Biomaghreb Laboratories (Tunisia). Enzymatic activity was expressed in international units per liter (IU/L). Additionally, we focused on measuring the activity of enzymes involved in hepatic metabolism to evaluate the effect of decavanadate accumulation on liver function. Creatinine level was expressed in mg/L.

#### 2.7.2. Histological Examination

Liver and kidney organs in formalin were washed and dehydrated using alcohol. Paraffin-embedded sections were cut at 5-6 μm thickness and stained with hematoxylin and eosin for microscopic examination at 20x and 40x magnification.

### 2.8. Experimental Design of the Chemical-Induced Melanoma Model in Mice

Two days before the beginning of the experiment, we selected 32 male Swiss albino mice and clipped the hair on their backs in a 2 × 2 cm^2^ area. The design of the experiment was performed as reported by Vähätupa et al. [63] (Figure S1). The animals were randomly divided into 3 groups (8 animals in each group):

i. CTR− group: represented the negative CTR group, where mice received 100 μL of acetone only.
ii. CTR+ group: served as the positive CTR group, where mice were treated subcutaneously with a single dose of DMBA (50 μg/100 μL acetone) on the shaven area on their backs. Seven days later, croton oil (50 μL/100 μL acetone) was applied twice a week until the end of the experiment (6 weeks).
iii. CT group (cotreatment group): animals received a single dose of DMBA (50 μg/100 μL acetone) on the shaven area on their backs. Seven days later, croton oil (50 μL/100 μL acetone) was applied twice. Then, mice received croton oil and 2.5 mg/kg of the decavanadate compound (dissolved in PBS) by subcutaneous injection on their backs. The Mg_2_Na_2_V_10_O_28_·20H_2_O compound was given to animals 15 min after each croton oil application from the third week until the sixth week (the end of the protocol).

### 2.9. Experimental Design of the Melanoma Allograft Model in Mice

The syngeneic transplantation model of melanoma, developed in this study, has been widely used for preclinical testing and offers the advantages of relative ease of use, fast turnaround time, and low cost. A total of 30 male C57BL/6 strain mice were previously adapted to animal facility conditions. The design of the experiment was performed as represented in Figure S2. At the start of the experiment, the backs of the mice were shaved. The mice were divided into four groups:

i. CTR− group: negative CTR group containing 6 mice. This group was injected with a solution of PBS by the subcutaneous route in the dorsal region.
ii. CTR+ group: positive CTR group containing 8 mice. In this group, the mice were subcutaneously injected once with 10^5^ B16-F10 cells in the dorsal region at the beginning of the experiment. Four mice were sacrificed after 21 days, and the other 4 mice were sacrificed at the end of the experiment after 31 days.
iii. Cotreatment (CT) group: CT group containing 8 mice. This group was subcutaneously injected with 10^5^ B16-F10 cells in the dorsal region and injected simultaneously with 2.5 mg/kg of decavanadate (Mg_2_Na_2_V_10_O_28_·20H_2_O), subcutaneously for 21 days of the experiment. All the mice were sacrificed after 21 days.
iv. Post-treatment (PT) group: PT group containing 8 mice. This group was subcutaneously injected with 10^5^ B16-F10 cells in the dorsal region. After 21 days, each mouse of this group received a dose of 2.5 mg/kg of decavanadate (Mg_2_Na_2_V_10_O_28_·20H_2_O), as a treatment for 10 days. For this group, animals were sacrificed on the 31st day. The experimental design scheme is illustrated in Figure S2.

### 2.10. Statistical Analysis

Statistical analysis was carried out with one-way ANOVA followed by a Dunnett test, using GraphPad Prism software (Version 8.4). Numerical results are from a representative experiment of at least three, expressed as mean ± standard error of the mean (SEM). Significance was set at the 95% level. Statistical significance levels were defined as ⁣^∗^*p* < 0.01, ⁣^∗∗^*p* < 0.001, and ⁣^∗∗∗^*p* < 0.0001. The IC_50_ values were calculated with regression analysis using the software GraphPad Prism Version 8.4 (GraphPad Software, San Diego, CA, USA) by fitting a variable slope-sigmoid dose–effect curve.

## 3. Results

### 3.1. Synthesis and Structural Characterization of Mg_2_Na_2_V_10_O_28_·20H_2_O

The decavanadate compound was obtained from a mixture of vanadium oxide (V_2_O_5_), magnesium hydroxide (Mg(OH)_2_·5H_2_O), and sodium hydroxide (NaOH) with molar proportions 5:2:2, as described in the Materials and Methods section. The solution of the decavanadate compound has a dark or light yellow color, depending on its concentration, and its pH is 6.

**Figure 1:**
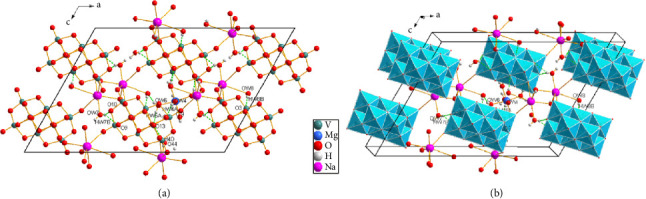
Representation of the 3D structure of the Mg_2_Na_2_V_10_O_28_·20H_2_O compound. (a) Projection of the structure according to *b*. (b) Perspective view of the structure built by the DIAMOND software [65].

After solving the structure, we determined the chemical formula of the synthesized phase of the Mg_2_Na_2_V_10_O_28_·20H_2_O compound. The research in the literature showed that the parameters of the lattice and the crystal system correspond to those published by Iida and Ozeki [64] with small differences in the cell parameters and the volume, with *a* = 23.8384(6) Å (vs. 24.545(5) Å); *b* = 11.0248(2) Å (vs. 10.913(3) Å); *c* = 16.9332(4) Å (vs. 17.586(4) Å); *β* = 118.005(1)° (vs. 119.50(5)°); and V = 3929.18(15) Å^3^ (vs. 4099.9(1) Å^3^). The structure obtained for the Mg_2_Na_2_V_10_O_28_·20H_2_O compound is shown in Figures 1(a) and 1(b). Crystallographic data and hydrogen bonds are given in Tables S1 and S2, respectively. We found that the decavanadate groups, sodium dimers, magnesium polyhedra, and water molecules are linked by oxygen vertices, hydrogen bonds, and van der Waals interactions, respectively. The crystallinity and purity of the prepared phase were checked by comparing the experimental diffractogram of the synthesized phase to the calculated (obtained from data from .cif files, using the POWDERCELL program [58]) diffractogram of the compound using the Origin 8.0 software [59].  Figure S3A shows that the angular positions of the lines in the experimental diffractogram are practically identical to those noted in the theoretical (calculated) diffractogram refined in the monoclinic system with the space group C2/c. This figure shows a good superposition of the experimental and theoretical diffractograms, with no additional peaks. All diffraction lines can be indexed in the same system with the same space group. This clearly indicates the purity of our phase.

**Figure 2:**
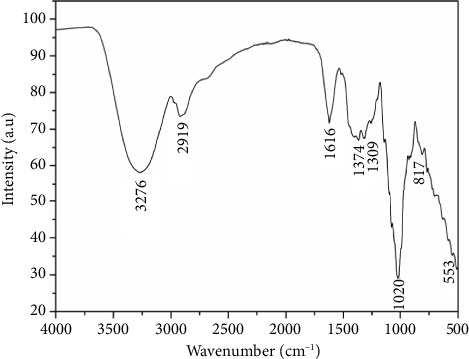
Infrared spectra of the Mg_2_Na_2_V_10_O_28_·20H_2_O compound.

The IR spectrum of the Mg_2_Na_2_V_10_O_28_·20H_2_O compound (Figure 2) shows a broad band between 3400 and 3000 cm^−1^ and a band around 2900 cm^−1^ that correspond to the vibration modes of water molecules and hydrogen bands. The elongation modes of water molecules are located around 1616 cm^−1^. The band located in 1300 cm^−1^ was attributed to the symmetric stretching vibrations of C=O in CO_2_ (g). The bands located at 1020, 817, and 553 cm^−1^ correspond to the vibration modes of the VO_6_ octahedral in the decavanadate group (Table S3). The TG-DTA curve of the compound Mg_2_Na_2_V_10_O_28_·20H_2_O (Figure S3B) showed a mass decrease corresponding to the loss of 20 water molecules (exp.: 28.57%; theo.: 28%). The second loss of mass corresponds to the departure of the inorganic part.

### 3.2. Decavanadate Specifically Affects the Viability of Melanoma Cells

**Figure 3:**
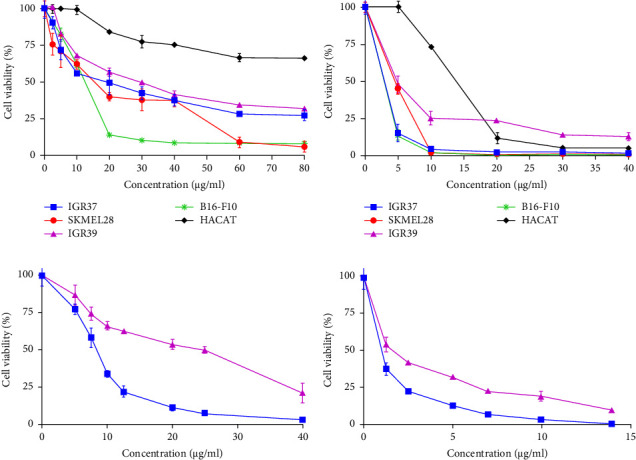
Effect of Mg_2_Na_2_V_10_O_28_·20H_2_O and cisplatin on the viability of different cutaneous cell lines. (a) Effect of Mg_2_Na_2_V_10_O_28_·20H_2_O on the viability of melanoma cells IGR37, SKMEL28, IGR39, and B16-F10 and on HaCaT normal keratinocytes after treatment for 24 h. (b) Effect of Mg_2_Na_2_V_10_O_28_·20H_2_O on the viability of melanoma cells IGR37, SKMEL28, IGR39, and B16-F10 and on HaCaT normal keratinocytes after treatment for 72 h. (c) and (d) Effect of cisplatin on human melanoma cells IGR37 and IGR39 after treatment for 24 h and 72 h, respectively. Cell viability was calculated as (*A*_560_ sample − *A*_560_ blank)/(*A*_560_ control − *A*_560_ blank). Blank corresponds to wells without cells. Dose–response plots (decavanadate (μM) versus cell viability (%)) and nonlinear regression were used to calculate IC_50_ values, using GraphPad Prism. The IC_50_ values are reported in Table 1.

Four cutaneous melanoma cell lines IGR39, IGR37, SKMEL28, and B16-F10 were treated with increasing concentrations of the decavanadate compound (Figure 3). A stock solution was prepared at a concentration of 1 mg/mL and kept at a temperature of +4°C. The dilutions were made just before the treatment. Figures 3(a) and 3(b) show the dose–response effect of Mg_2_Na_2_V_10_O_28_·20H_2_O compound on the viability of different melanoma cell lines, after 24 and 72 h of treatments, respectively. Results showed that after 24 h, the Mg_2_Na_2_V_10_O_28_·20H_2_O compound significantly reduced the viability of all the tested cell lines, in a dose-dependent manner with IC_50_ values varying between 10.3 μg/mL (7.3 μM) and 25.2 μg/mL (18 μM) (Figure 3(a) and Table 1). Interestingly, the effect was more important after 72 h of treatment, where the decavanadate compound significantly increased the cell death with IC_50_ values ranging from 2 μg/mL (1.4 μM) to 7.4 μg/mL (5.2 μM) (Figure 3(b) and Table 1). The best IC_50_ value of 2 μg/mL (1.4 μM) was obtained against the human IGR37 cell line. The IC_50_ value on the human normal keratinocyte cells (HaCaT) was 12.7 μg/mL (8.9 μM), after 72 h of treatment (Figure 3(b)), while little effect was observed after 24 h of treatment (Figure 3(a)).

It is worth noting that when the experiments are repeated after 2 months from the same stock solution, the decavanadate remains active and we get the same results, showing that the compound is stable.

We then tested cisplatin, a chemotherapeutic agent that generates DNA damage and induces cell apoptosis, commonly used in the treatment of malignant melanoma. We found that cisplatin displayed IC_50_ values of 25 μg/mL (83 μM) and 8 μg/mL (26.5 μM) against human IGR39 and IGR37 cell lines, respectively, after 24 h (Figure 3(c)). These values decreased to 3.8 μg/mL (12.5 μM) and 0.9 μg/mL (3 μM), respectively, after 72 h (Figure 3(d)).

### 3.3. Decavanadate Affects Adhesion and Migration of IGR39 Human Melanoma Cells

**Figure 4:**
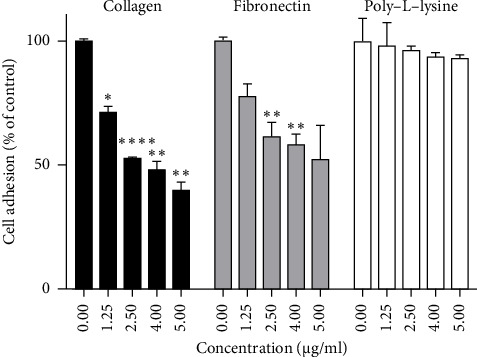
Effect of Mg_2_Na_2_V_10_O_28_·20H_2_O on IGR39 cell attachment to fibronectin, collagen, and poly-L-lysine using cell adhesion assay. IGR-39 human melanoma cells were pretreated with PBS (0.00) or with different concentrations of Mg_2_Na_2_V_10_O_28_·20H_2_O (1.25–5.00 μg/mL) for 30 min. They were then allowed to adhere for 1 h to wells coated with 10 μg/mL of collagen or fibronectin, or with the nonspecific substratum poly-L-lysine at 50 μg/mL. Adherent cells were labeled with crystal violet, and absorbance was measured at 600 nm. Data are reported as means ± SEM of three separate experiments. (*N* = 3) (⁣^∗^*p* < 0.01, ⁣^∗∗^*p* < 0.001, and ⁣^∗∗∗^*p* < 0.0001, with respect to nontreated cells as control).

In order to check the effect of the decavanadate compound on malignant transformation, cancer progression, and metastasis, we first performed a cell adhesion assay using collagen and fibronectin as ECM, as well as poly-L-lysine, as a nonspecific attachment substrate for cells. We have chosen to study the IGR39 cells because Mg_2_Na_2_V_10_O_28_·20H_2_O was less cytotoxic on this cell line. As illustrated in Figure 4, the decavanadate compound was efficient in decreasing IGR39 cell attachment to collagen in a dose-dependent manner, with an IC_50_ value of 3.78 μg/mL (2.67 μM). The reduction reached 60% with the highest concentration of 5 μg/mL (3.5 μM). The decavanadate compound had a lesser effect (47%) in inhibiting the adhesion of the IGR39 cells on the fibronectin at 5 μg/mL, while no effect was observed on poly-L-lysine until this concentration (Figure 4).

**Figure 5:**
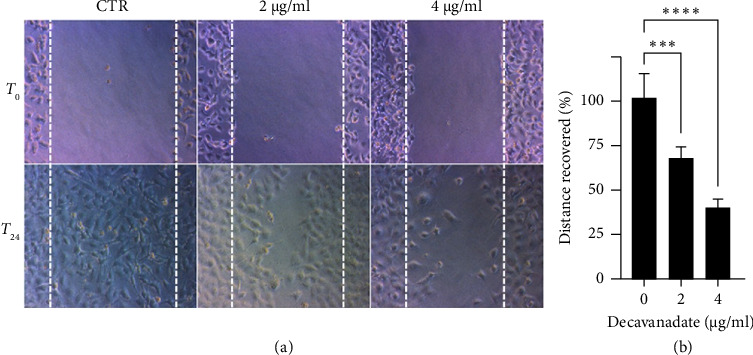
Effect of Mg_2_Na_2_V_10_O_28_·20H_2_O on the migration of IGR39 human cutaneous melanoma cells by the wound healing test. (a) Microscopic images of the wounds before migration (*T*_0_) and after 24 h (*T*_24_) migration of IGR39 cells untreated (CTR) or treated with 2 μg/mL (1.4 μM) or 4 μg/mL (2.8 μM) of Mg_2_Na_2_V_10_O_28_·20H_2_O compound. (b) Representative histograms of cell migration (in %) after treatment by Mg_2_Na_2_V_10_O_28_·20H_2_O. The percentage of cell migration was calculated using the following formula [(*T*_0_ − *T*_24_))/(*T*_0_)] × 100 where *T*_0_ is the wound area at zero time and *T*_24_ is the wound area after 24 h. Data were analyzed using a two-way ANOVA test (Prism 9 software [66]) to evaluate the significance between three independent tests. To be considered representative, each condition was compared with control using a *p* value < 0.05 (⁣^∗∗∗^*p* < 0.001; ⁣^∗∗∗∗^*p* < 0.0001).

During metastasis, cancer cells spread from their primary site to the surrounding tissue and invade nearby and distant organs. Stopping or reducing cell migration and infiltration is essential for developing novel therapeutic approaches to treat melanoma. In this context, the effect of the decavanadate compound on IGR39 cell motility was evaluated using the wound-healing assay. Thus, cell migration was assessed upon treatment with the decavanadate compound at subcytotoxic concentrations of 2 μg/mL (1.4 μM) and 4 μg/mL (2.8 μM). As shown in Figure 5, the gap closure, corresponding to the cell migration, was significantly impaired after 24 h of treatment when compared to untreated CTR cells. The effect of Mg_2_Na_2_V_10_O_28_·20H_2_O on IGR39 cell migration was dose-dependent with an IC_50_ value of 3.16 μg/mL (2.23 μM).

Our findings showed that the decavanadate compound was able to block IGR39 melanoma cell adhesion and migration.

### 3.4. Assessment of Toxic/Safe Doses of Decavanadate

We first checked the safe dose of Mg_2_Na_2_V_10_O_28_·20H_2_O for its possible therapeutic use. The stock solution was prepared at a concentration of 7.5 mg/mL in PBS (1x) and kept at a temperature of +4°C. Dilutions were freshly prepared and injected into mice.

No mortality or signs of toxicity, including weight loss or behavioral abnormalities, were noticed in mice injected with 1 mg/kg (20 μg per mouse), 2.5 mg/kg (50 μg per mouse), or 5 mg/kg (100 μg per mouse) of Mg_2_Na_2_V_10_O_28_·20H_2_O during all the treatments. After 12 days, the blood and organs of mice were examined.

The evaluation of biochemical parameters in blood sera showed that the transaminase (AST and ALT) activities as well as the creatinine levels increased significantly in mice receiving 5 mg/kg of the decavanadate compound. However, no significant difference was noticed in the transaminase activities of mice receiving 2.5 mg/kg of the compound, while their creatinine level increased to 2.45 mg/L, compared to those of the negative CTR group (1.57 mg/L) (Table 2). For the dose of 1 mg/kg, all the parameters were comparable to the negative CTR and no significant differences were detected.

**Figure 6:**
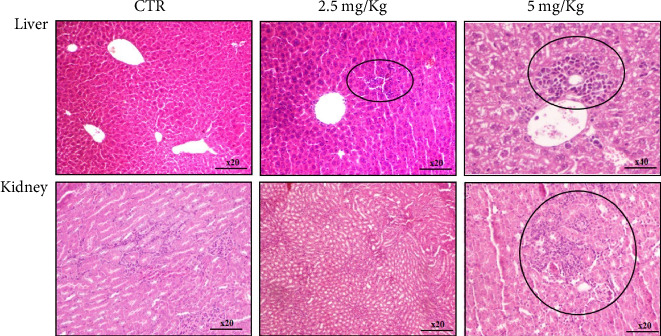
Histological study of the liver (up) and kidney sections of the CTR group, the group treated with 2.5 mg/kg, and the group treated with 5 mg/kg of Mg_2_Na_2_V_10_O_28_·20H_2_O. The sections were stained with hematoxylin and eosin. The circles highlight the altered tissue.

Liver histological analysis showed necrosis in mice treated with 50 μg (2.5 mg/kg) of the decavanadate compound and a lymphohistiocytic inflammatory infiltrate in the portal tract in mice treated with the dose of 100 μg (5 mg/kg), when compared to normal tissue architecture in mice liver of the CTR group (Figure 6). The kidney sections of the group treated by 2.5 mg/kg of Mg_2_Na_2_V_10_O_28_·20H_2_O compound displayed no difference compared to those of the CTR group, while the tissue architecture of mice injected with 5 mg/kg of decavanadate was altered with a mild interstitial lymphocytic inflammatory infiltrate (Figure 6). Thus, all these results showed that Mg_2_Na_2_V_10_O_28_·20H_2_O is nephrotoxic at the dose of 5 mg/kg and that the dose of 2.5 mg/kg could be considered relatively safe although there is slight toxicity in the liver. Thus, we chose the dose of 2.5 mg/kg to study its potential antimelanoma effect in vivo.

### 3.5. Decavanadate Attenuates Inflammation and Tumor Development in Mice

In order to assess the antitumor effect of the decavanadate compound on melanoma in mice, we developed two different melanoma models: (i) chemically induced lesion model and (ii) mouse allograft model.

**Figure 7:**
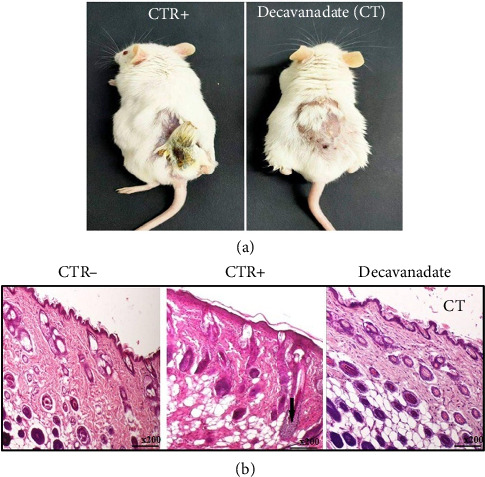
(a) Illustration of the difference in the lesions appearance between the CTR+ group and the group cotreated with decavanadate (CT). (b) Histology of skin tissues corresponding to the normal CTR− group, the CTR+ group, showing dense dermal lymphohistiocytic infiltrate (arrow), and the group of mice cotreated with decavanadate.

After being treated with DMBA/croton oil (CTR+ group), the mice showed hypoactivity and paralysis in the back legs during the 6 weeks of the experiment and the mortality rate reached 50% in this group. Interestingly, neither mortality nor changes in the behavior were noticed in mice injected with 2.5 mg/kg of decavanadate along with DMBA/croton oil (cotreatment group), as well as in those of the CTR− group. On the other hand, as shown in Figure 7(a), we observed a notable difference in the evolution of the lesions as well as their appearance, between CTR+ and cotreatment groups (Figure 7(a)). These results showed that the Mg_2_Na_2_V_10_O_28_·20H_2_O compound protected animals from the toxic effects of DMBA/croton oil.

Histological analysis showed that the vehicle injection did not alter the skin of the CTR− group mice. However, the skin of the CTR+ group shows the appearance of dermal polymorphic inflammatory infiltrate (Figure 7(b)). Interestingly, the skin of mice cotreated with decavanadate showed an attenuation of this inflammatory state (Figure 7(b)).

In the second model consisting of mice allografted with B10-F10 cells (CTR+ group), we found that the mice exhibited hypoactivity and hindlimb paralysis, as previously observed with DMBA/croton oil injection, but no mortality was observed in any group. Interestingly, no change in the behavior was noticed in mice cotreated with the Mg_2_Na_2_V_10_O_28_·20H_2_O compound, compared to those of the CTR− group.

**Figure 8:**
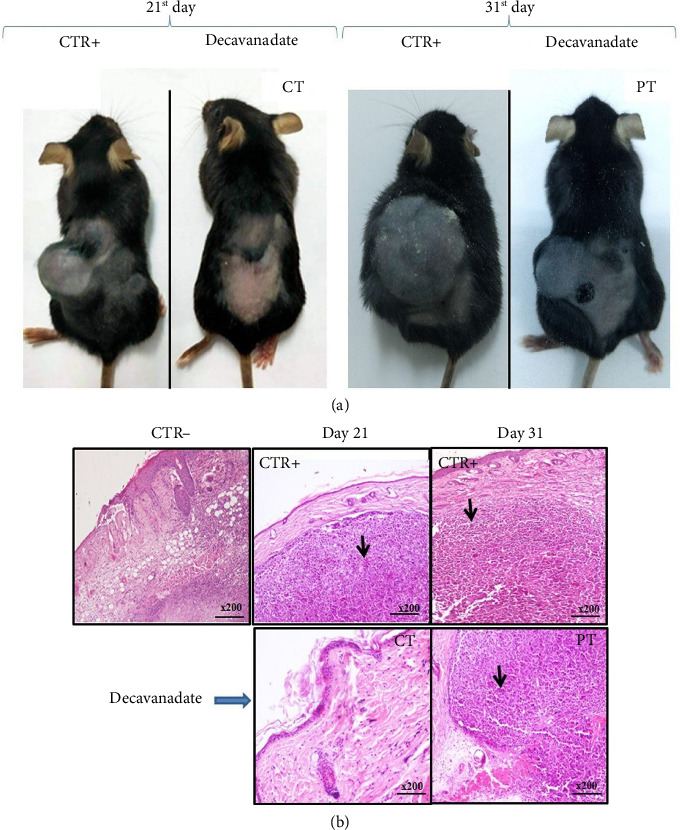
(a) Photograph of a representative mouse of the CTR+ group showing the appearance of dermal melanoma and those of groups of cotreated (CT) and post-treated (PT) mice with the decavanadate compound. (b) Skin histology of the CTR− group, the CTR+ group and decavanadate-treated groups: post-treated (PT) and cotreated (CT). The CTR+ and post-treated groups with the decavanadate compound show the appearance of dermal melanoma associated with invasive nests of atypical discohesive melanocytes and dusty pigmentation (shown by arrow).

Twenty-one days after subcutaneous injection of B16-F10 melanoma cells, we observed a tumor of 12.7 mm in average size in mice of the CTR+ group, while those cotreated with the Mg_2_Na_2_V_10_O_28_·20H_2_O compound showed no skin changes (Figure 8(a)). After 31 days, the average size of the tumor reached 27.3 mm in mice of the CTR+ group and 8.2 mm in mice of the PT group (after the 21st day) with the decavanadate compound. Thus, treatment of allografted mice with the Mg_2_Na_2_V_10_O_28_·20H_2_O compound inhibited tumor development by 70%.

Histological analysis of the CTR + group shows the appearance of dermal melanoma associated with invasive nests of atypical discohesive melanocytes and a dusty pigmentation. Melanoma persists in the PT group. As expected, melanoma did not appear in the CT group (Figure 8(b)).

## 4. Discussion

Many studies showed that decavanadates have more powerful biological activities than other POM compounds [67]. In our previous studies, we synthesized and characterized several decavanadate compounds [30, 31, 35–54]. The structure and activity characterization of these compounds allowed us to identify structural elements likely to confer high stability and/or better activity to the decavanadate compounds.

Based on these studies, we designed and synthesized the Mg_2_Na_2_V_10_O_28_·20H_2_O and investigated its efficiency against the development of different human and murine melanoma cell lines, as well as on chemical-induced and allografted tumors on mice models, with the aim to validate its efficiency both in vitro and in vivo.

The structure of the Mg_2_Na_2_V_10_O_28_·20H_2_O compound was determined by X-ray crystallography and characterized by several spectroscopic techniques, showing that the crystal is structured in the monoclinic system with the space group C2/c and clearly indicated the purity of our phase. As expected, we found that the sodium polyhedra form oxygen bridges with the decavanadate groups giving a great stability to the structure of the Mg_2_Na_2_V_10_O_28_·20H_2_O compound. The stability of the compound is further improved by the 20 water molecules, thanks to the O-H…O hydrogen-bond type.

Furthermore, our compound had an acidic character since its pH was 6. In addition, until a temperature of 100°C, we have not seen any loss of mass (Figure S3B), as shown for other POMs [68]. All these results evidenced the stability of the Mg_2_Na_2_V_10_O_28_·20H_2_O compound. In fact, it was reported that the speciation of POMs is heavily influenced by pH, which affects their formation and stability in solution. POMs tend to be more stable in acidic environments, with their resistance to hydrolysis increasing as their negative charge grows. Temperature also impacts speciation, with unstable POMs like Keggin-type PW12 hydrolyzing even at room temperature, while decavanadate compounds did not display any noticeable changes in stability when they were exposed to elevated temperatures (37°C) [68, 69]. The stability of POMs is a critical factor when considering their application, especially in biological systems.

Because cancer is characterized by several hallmarks including cell proliferation in an anarchic way [70], we studied the effect of the Mg_2_Na_2_V_10_O_28_·20H_2_O on human and murine melanoma cell growth. We found that it inhibited their growth in a dose-dependent manner. Interestingly, this decavanadate compound exhibited higher effect after treatment of 72 h than 24 h, showing that it is rather active on the proliferation of these cell lines.

More interestingly, Mg_2_Na_2_V_10_O_28_·20H_2_O displayed a pronounced cytotoxic effect on the four cutaneous melanoma cell lines IGR39, IGR37, SKMEL28, and B16-F10 (with IC_50_ values ranging from 7.3 to 18 μM), while having only a little effect on normal human keratinocyte cells HaCaT (IC_50_ > 70 μM). This suggests that the cytotoxic effect of the decavanadate compound is exerted preferentially on tumor cells rather than on normal cells.

Interestingly, the decavanadate is more active than the chemotherapeutic agent cisplatin on IGR37 and IGR39 human melanoma cells. This molecule was reported to have IC_50_ values >> 12.5 μM and equal to 9.5 μM on human SK-MEL-28 melanoma, for 24 h and 72 h of treatments, respectively [71]. These IC_50_ values are 19.5 and 10 μM on murine B16-F10 melanoma cells [72, 73]. In fact, the concentration of cisplatin could be compared to that of Mg_2_Na_2_V_10_O_28_·20H_2_O in terms of molarity, even if there is only one atom of Pt in cisplatin, whereas there are 10 atoms of vanadium in decavanadate. It is noteworthy that the chemotherapeutic agent displayed an IC_50_ value of 25 μM against normal human keratinocyte cells [74], thus clearly showing the advantage of the Mg_2_Na_2_V_10_O_28_.20H_2_O compound over cisplatin which was notably very toxic for normal keratinocytes.

Other decavanadate compounds have been tested on different melanoma cell lines. For instance, metformin–decavanadate (Metf-V_10_) inhibited by 80% the proliferation of UACC-62 human melanoma cells, at 5 μM [33]. This effect is comparable to that of our compound. On the other hand, our compound showed a lesser effect on the proliferation of IGR39 than Mg(H_2_O)_6_(C_4_N_2_H_7_)_4_V_10_O_28_·4H_2_O (IC_50_ = 2 μM) [31], while it was more active than the (C_4_N_2_H_7_)_6_(C_4_N_2_H_6_)_2_V_10_O_28_ (IC_50_ = 7 μM) [53] and (NH_4_)_4_Li_2_V_10_O_28_10H_2_O compounds (IC_50_ = 9.8 μM) [30], both synthesized by our group.

When compared to other chemical and/or natural molecules having antimelanoma effects, we can consider that our Mg_2_Na_2_V_10_O_28_·20H_2_O has a potent antitumor activity. In fact, rutoside (a natural flavonoid) displayed an IC_50_ value of about 100 μg/mL (163 μM) on A375 human melanoma cell viability after 24 h of treatment [75]. Minocycline and doxycycline, two antibiotics from the tetracycline family, inhibited COLO829 melanoma cell proliferation with IC_50_ values of 13.9 and 16.25 μM, respectively [76]. It should be noted that rutoside is a potential drug for various disorders and has been used for its special anticancer effects [77]. It was reported to be a useful adjunct to radiotherapy and chemotherapy, notably in combination with other drugs [78].

Although the mechanism of action of decavanadate complexes as antitumor agents is not yet fully understood, the decavanadate effects on tumor proliferation might, in part, be due to the inhibition of certain enzymes such as alkaline phosphatases and ecto-nucleotidases as well as ATPases [21]. Indeed, decavanadate species were described as the only identified POVs that inhibit calcium accumulation by the Ca^2+^-ATPase when coupled to ATP hydrolysis [79]. In addition, V10 appears to be the most potent oxometalate Ca^2+^-ATPase inhibitor out of all the vanadium ions and complexes so far analyzed, exhibiting an IC_50_ value of 15 μM [79, 80]. Modulation of expression of a plasma membrane Ca^2+^-ATPase (PMCA) isoform in cancer cells has been shown to provide an advantage over growth by increasing responses to proliferative stimuli or reducing sensitivity to apoptosis [81]. PMCA pumps are key regulators of cytosolic Ca^2+^ in eukaryotes. They extrude Ca^2+^ from the cytosol, using the energy of ATP hydrolysis, and operate as Ca^2+^-H^+^ exchangers. They are activated by the Ca^2+^-binding protein calmodulin, by acidic phospholipids, and by other mechanisms, among them kinase-mediated phosphorylation [82].

This can explain our results, showing that the IGR37 cell line was the most sensitive toward the Mg_2_Na_2_V_10_O_28_·20H_2_O, while the IGR39 cell line was the least sensitive. In fact, IGR39 and IGR37 cell lines have been established from the primary and metastatic tumors respectively, of the same patient. However, the total calmodulin level was found to be 2 to 4 times higher in IGR37 than in the IGR39 cells [83]. Thus, the most plausible hypothesis is that the high sensitivity of the IGR37 cell line to the decavanadate molecule, compared to the primary melanoma cells IGR39, is the consequence of the interaction of Mg_2_Na_2_V_10_O_28_·20H_2_O with ATPases, inhibiting its activity, controlled by calmodulin modulation [84, 85].

One of the threatening capacities of cancer cells is metastasis through their ability to spread throughout different tissues using attachment to ECM and cell migration capabilities [86, 87]. Adhesion is a crucial step in cancer progression, as it allows cancer cells to anchor themselves firmly, promoting their survival, proliferation, and invasion into surrounding tissues. Interestingly, we found that the Mg_2_Na_2_V_10_O_28_·20H_2_O compound was able to reduce the ability of IGR39 melanoma cells to adhere to both ECM, collagen and fibronectin, with a more pronounced effect on collagen. However, it did not affect the adhesion of melanoma cells to poly-L-lysine, suggesting that the observed effect on cell adhesion is related to integrins. These are the major receptors for ECM proteins that play a key role in the cell adhesion process and are also involved in cell proliferation and migration [88]. As expected, at 2 μg/mL, Mg_2_Na_2_V_10_O_28_·20H_2_O was also able to inhibit the migration of IGR39 cells. The effect of Mg_2_Na_2_V_10_O_28_·20H_2_O on IGR39 cell adhesion and migration may be due to the inhibition of the *α*2*β*1 integrin, since this integrin is reported to be highly expressed in IGR39 cells, contributing to their adhesion and invasion process [89].

As our in vitro study showed that Mg_2_Na_2_V_10_O_28_·20H_2_O presented a powerful effect in B16-F10 murine melanoma cells, we used the nontoxic dose of 2.5 mg/kg (50 μg/mouse) to investigate its in vivo effect on the mice induced melanoma models. Our results showed that the cotreatment by Mg_2_Na_2_V_10_O_28_·20H_2_O attenuated the level of inflammation, associated with the decrease in the infiltration of lymphocytes, caused by the subcutaneous injection of DMBA and croton oil [60]. Interestingly, we found that Mg_2_Na_2_V_10_O_28_·20H_2_O was more potent than the orthovanadate, which partially inhibited the incidence of skin papillomas caused by the injection of DMBA and croton oil at 5 mg/kg [90].

Furthermore, our results showed that Mg_2_Na_2_V_10_O_28_·20H_2_O cotreatment hinders the growth of B16-F10-induced tumors in allografted mice. This is confirmed by histological analysis, showing the disappearance of melanoma in tissues of cotreated mice, compared to those of CTR. Our results are in harmony with those reported by Ferreira et al. and Thompson et al., showing that vanadate and vanadyl sulfate, respectively, significantly decreased tumor size and their incidence in an allograft model of B16-F10 cells in mice [91, 92].

## 5. Conclusion

In light of these results, we can conclude that the Mg_2_Na_2_V_10_O_28_·20H_2_O compound has a stable structure, in which the vanadium forms complexes with water molecules and magnesium and sodium atoms, taking a structure that allows an efficient biological activity [93]. Indeed, compared to reported molecules having effects on melanoma, the Mg_2_Na_2_V_10_O_28_·20H_2_O compound could be considered a potent agent, able to prevent the development and metastasis of melanoma, one of the most invasive cancers. The Mg_2_Na_2_V_10_O_28_·20H_2_O compound displayed an advantage over cisplatin, the chemotherapy agent, which was shown to be much more aggressive to the normal keratinocyte cells.

It would be interesting to investigate the mechanism of action of Mg_2_Na_2_V_10_O_28_·20H_2_O on melanoma cells, especially the apoptotic pathway involving the calcium/calmodulin kinase II complex (CaMKII), which controls and inhibits the affinity state of many integrins [94, 95], and to have a detailed knowledge of the molecular basis and cellular interactions in order to understand the processes associated with its promising anticancer applications.

## Figures and Tables

**Table 1:** IC_50_ values (μM) of Mg_2_Na_2_V_10_O_28_·20H_2_O and cisplatin on different melanoma cell lines and human normal keratinocytes.

Treatment duration (h)	Cell line molecule	IGR37	SKMEL28	IGR39	B16-F10	HaCaT
24	Mg_2_Na_2_V_10_O_28_·20H_2_O	12.7	10.8	18	7.3	> 70.8
	Cisplatin	26.5	> 12.5 [35]	83	19.5 [36]	23.9 [38]

72	Mg_2_Na_2_V_10_O_28_·20H_2_O	1.4	4.1	5.2	2.8	8.9
	Cisplatin	3	9.5 [35]	12.5	10 [37]	23.9 [38]

**Table 2:** Effect of the decavanadate compound on hepatic and renal functions in mice.

Groups	ALT (IU/L)	AST (IU/L)	Creatinine (mg/L)
CTR	42.87 ± 3.2		1.57 ± 0.22
2.5 mg/kg	48.75 ± 2.96	32.83 ± 1.09	2.45 ± 0.28⁣^∗^
5 mg/kg	81.67 ± 5.12⁣^∗∗∗^	45.63 ± 1.21⁣^∗∗^	2.80 ± 0.312⁣^∗∗^

Note: The amounts of ALT and AST are expressed in IU/L. Statistical analysis was performed by ANOVA. Comparison of control and other groups: ⁣^∗^*p* < 0.05, ⁣^∗∗^*p* < 0.01, and ⁣^∗∗∗^*p* < 0.001.

## Data Availability

All data supporting the results are included within the article and in the Supporting Information.

## Supporting Information

Supporting Information 1 The following supporting information can be downloaded at https://www.mdpi.com/xxx/s1, Figure S1: Experimental design of the chemical-induced melanoma model in mice.

Supporting Information 2 Figure S2: Experimental design of the melanoma allograft model in mice.

Supporting Information 3 Figure S3: Analytical characterization of the Mg_2_Na_2_V_10_O_28_·20H_2_O compound structure: (A) Superposition of experimental and theoretical X-ray powder diffraction patterns; (B) TG-DTA analysis data.

Supporting Information 4 Table S1: X-ray crystallographic data of Mg_2_Na_2_V_10_O_28_·20H_2_O compound.

Supporting Information 5 Table S2: Hydrogen bonds of Mg_2_Na_2_V_10_O_28_·20H_2_O compound.

Supporting Information 6 Table S3: IR.
