# Supplementary figures and images for: Decavanadate Compound Displays In Vitro and In Vivo Antitumor Effect on Melanoma Models
Source: Bioinorg Chem Appl. 2025 Jan 11;2025:6680022. doi: 10.1155/bca/6680022 (PMC11742080; doi:10.1155/bca/6680022)

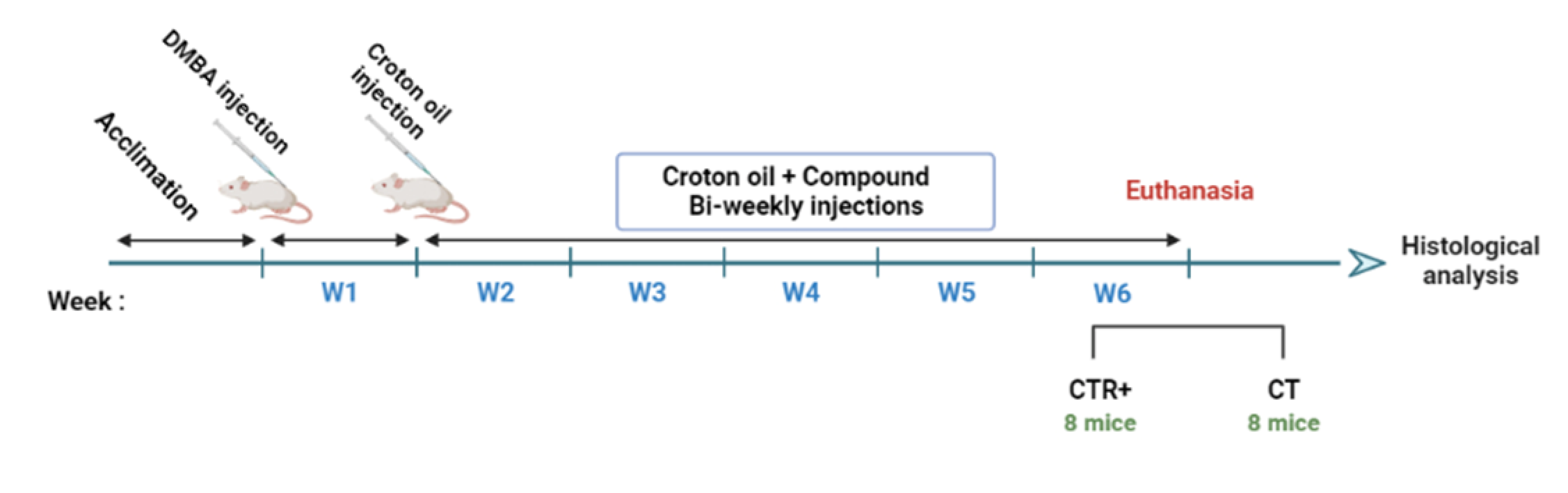

Supplement: Supporting Information 1 — The following supporting information can be downloaded at https://www.mdpi.com/xxx/s1, Figure S1: Experimental design of the chemical-induced melanoma model in mice. [file 6680022.f1.tif]

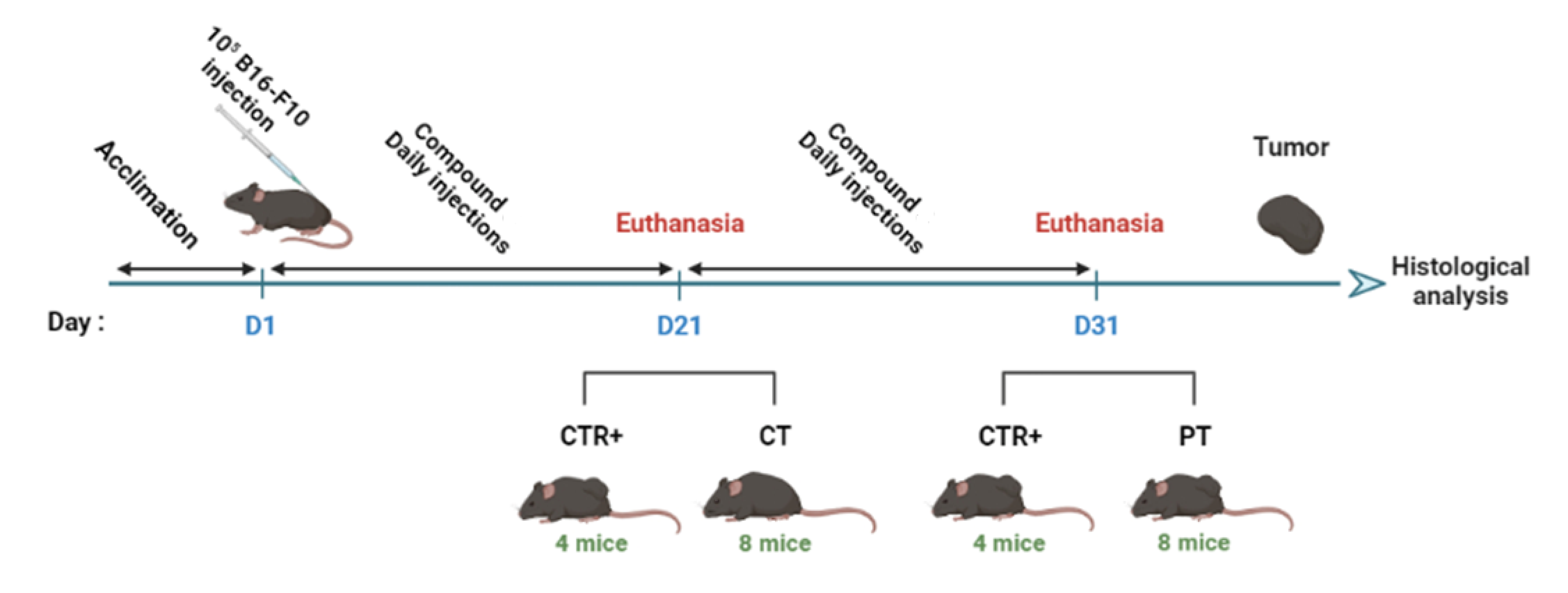

Supplement: Supporting Information 2 — Figure S2: Experimental design of the melanoma allograft model in mice. [file 6680022.f2.tif]

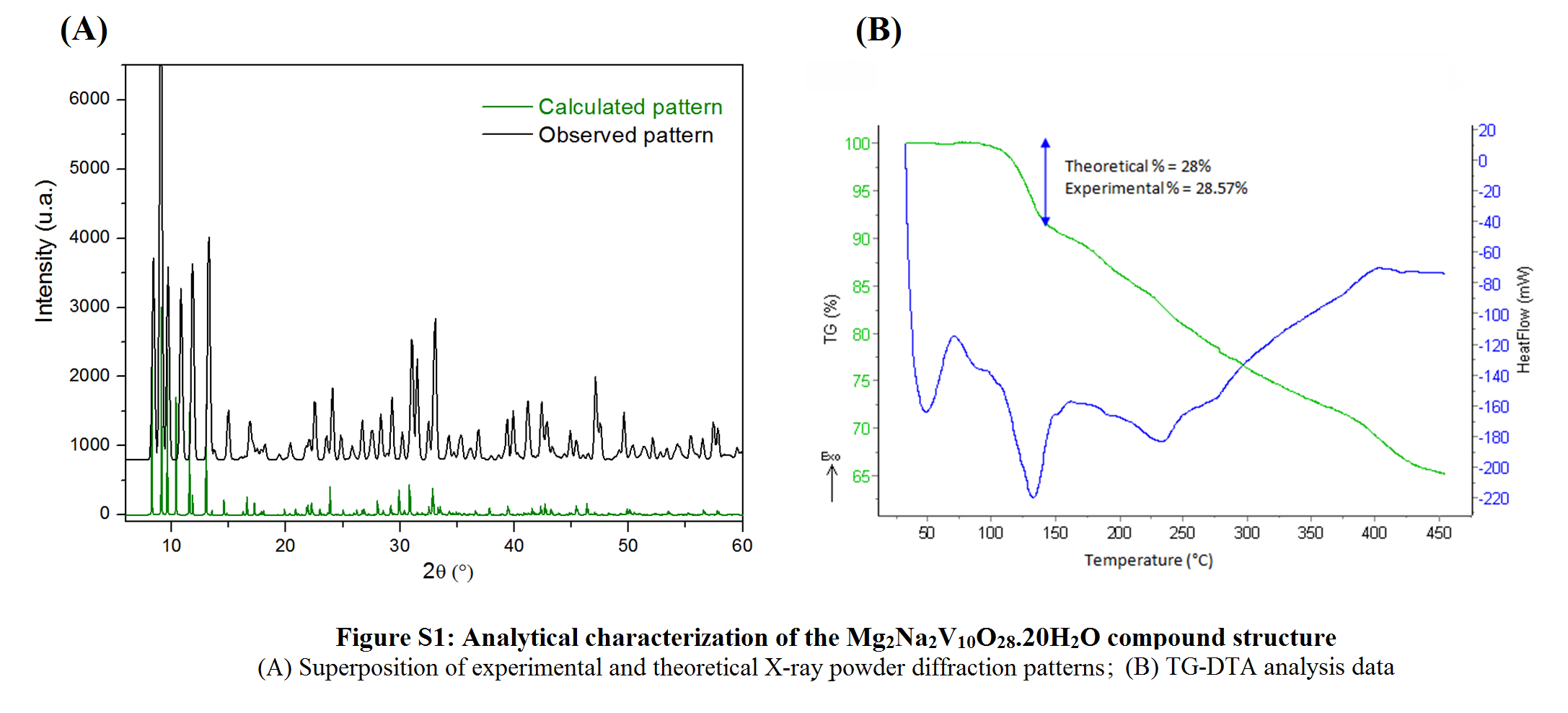

Supplement: Supporting Information 3 — Figure S3: Analytical characterization of the Mg2Na2V10O28·20H2O compound structure: (A) Superposition of experimental and theoretical X-ray powder diffraction patterns; (B) TG-DTA analysis data. [file 6680022.f3.tiff]
