# Supplementary material for: Decavanadate Compound Displays In Vitro and In Vivo Antitumor Effect on Melanoma Models
Source: Bioinorg Chem Appl. 2025 Jan 11;2025:6680022. doi: 10.1155/bca/6680022 (PMC11742080; doi:10.1155/bca/6680022)
Supplement: Supporting Information 4 — Table S1: X-ray crystallographic data of Mg2Na2V10O28·20H2O compound. [file 6680022.f4.docx]

**Table S1: X-ray crystallographic data of Mg_2_Na_2_V_10_O_28_.20H_2_O compound**

| **Crystal data** | |
| --- | --- |
| Chemical formula | H_44_Mg_2_Na_2_O_48_V_10_ |
| *M*_r_ | 1416.35 |
| Crystal system, space group | Monoclinic, *C*2/*c* |
| Temperature (K) | 293 |
| *a*, *b*, *c* (Å) | 24.545 (5), 10.913 (3), 17.586 (4) |
| β (°) | 119.50 (5) |
| *V* (Å^3^) | 4099.9 (1) |
| *Z* | 4 |
| Radiation type | Mo *K*α |
| µ (mm^−1^) | 2.36 |
| Crystal size (mm) | 0.14 x0.28 x0.45 |
|  | |
| **Data collection** | |
| Diffractometer | Enraf-Nonius CAD4 |
| Absorption correction | Ψscan |
| No. of measured, independent and observed [*I* > 2σ(*I*)] reflections | 4689, 4105, 3214 |
| *R*_int_ | 0.053 |
| (sin θ/λ)_max_ (Å^−1^) | 0.620 |
|  | |
| Refinement | |
| *R*[*F*^2^ > 2σ(*F*^2^)], *wR*(*F*^2^), *S* | 0.060, 0.174, 1.08 |
| No. of reflections | 4105 |
| No. of parameters | 351 |
| No. of restraints | 21 |
| H-atom treatment | Only H-atom coordinates refined |
|  | *w* = 1/[σ^2^(*F*_o_^2^) + (0.1064*P*)^2^ + 18.680*P*] where *P* = (*F*_o_^2^ + 2*F*_c_^2^)/3 |
| Δρ_max_, Δρ_min_ (e Å^−3^) | 1.32, −1.80 |
