# Supplementary material for: Decavanadate Compound Displays In Vitro and In Vivo Antitumor Effect on Melanoma Models
Source: Bioinorg Chem Appl. 2025 Jan 11;2025:6680022. doi: 10.1155/bca/6680022 (PMC11742080; doi:10.1155/bca/6680022)
Supplement: Supporting Information 5 — Table S2: Hydrogen bonds of Mg2Na2V10O28·20H2O compound. [file 6680022.f5.docx]

**Table S2: Hydrogen bonds of Mg_2_Na_2_V_10_O_28_.20H_2_O compound**

| **D-H···A** | **D (D-H) (Å)** | **d (H···A) (Å)** | **d (D···A) (Å)** | **<D-H···A> (°)** |
| --- | --- | --- | --- | --- |
| **OW1-HW1A...OW6^i^** | **0.892** | **1.910** | **2.795** | **139.97** |
| **OW1-HW1B...O2** | **0.905** | **1.732** | **2.634** | **174.29** |
| **OW2-HW2A...O6^ii^** | 0.901 | 1.827 | 2.724 | 173.33 |
| OW2-HW2B...OW8^i^ | 0.82 | 1.892 | 2.71 | 175.24 |
| OW3-HW3A...OW9^iii^ | 0.755 | 1.986 | 2.737 | 172.96 |
| **OW3-HW3B...OW7** | 0.771 | 1.980 | 2.77 | 166.22 |
| OW8-HW4B...O4^iv^ | 1.160 | 2.553 | 3.679 | 163.15 |
| OW8-HW4B…O8^iv^ | 1.160 | 2.652 | 3.581 | 136.23 |
| OW5-HW5A…O10^vi^ | 0.888 | 2.036 | 2.922 | 174.95 |
| OW5-HW5B… O7 | 0.892 | 1.962 | 2.820 | 161.04 |
| OW6-HW6A… O13^vii^ | 0.898 | 2.296 | 2.958 | 130.36 |
| OW6-HW6A… O14^viii^ | 0.898 | 2.258 | 2.983 | 137.59 |
| OW6-HW6B… OW8 | 0.896 | 1.885 | 2.761 | 165.09 |
| OW7-HW7A… OW9 | 0.890 | 1.922 | 2.811 | 177.09 |
| OW7-HW7B… O9^vii^ | 0.893 | 2.046 | 2.837 | 146.95 |
| OW7-HW7B… O10^vii^ | 0.893 | 2.659 | 3.423 | 144.14 |
| O44-H4B…O8^vii^ | 1.228 | 2.578 | 3.637 | 143.31 |
| O44-H4C… OW5^ii^ | 0.895 | 2.426 | 3.112 | 133.67 |
| OW8-HW8A… O1 | 0.886 | 2.020 | 2.905 | 177.59 |
| OW8-HW8B… O3^iv^ | 0.882 | 1.887 | 2.729 | 158.94 |
| OW9-HW9A… O6^vi^ | 0.890 | 2.049 | 2.800 | 141.43 |
| OW9-HW9B… OW7 | 0.887 | 2.108 | 2.811 | 135.65 |

Symmetry codes: i: -x+1, -y, -z+1, ii: x, -y, z+1/2, iii : -x+3/2, y-1/2, -z+3/2, iv^i^: -x+1, -y+1, -z+1, vi: -x+3/2, -y+1/2, -z+1, vii: x, -y+1, z+1/2 ; viii: x, -y, z+1/2
