# Supplementary material for: Decavanadate Compound Displays In Vitro and In Vivo Antitumor Effect on Melanoma Models
Source: Bioinorg Chem Appl. 2025 Jan 11;2025:6680022. doi: 10.1155/bca/6680022 (PMC11742080; doi:10.1155/bca/6680022)
Supplement: Supporting Information 6 — Table S3: IR. [file 6680022.f6.docx]

**Table S3. Assignments of the IR wave-numbers for Mg_2_Na_2_V_10_O_28_^.^20H_2_O**

| **Infrared (cm^-1^)** | **Assignments** |
| --- | --- |
| 3400-3000 | ν_OH_(H_2_O) |
| 2900 | ν(O-H…O) |
| 1616 | δ_OH_(H_2_O) |
| 1300 | ν_s_(C=O) |
| 1020 | ν(V=O) |
| 817 | ν_a_(O-V-O) |
| 553 | ν_s_(O-V-O) |
